# The Human Papillomavirus (HPV) E1 protein regulates the expression of cellular genes involved in immune response

**DOI:** 10.1038/s41598-019-49886-4

**Published:** 2019-09-20

**Authors:** Leonardo Josué Castro-Muñoz, Joaquín Manzo-Merino, J. Omar Muñoz-Bello, Leslie Olmedo-Nieva, Alberto Cedro-Tanda, Luis Alberto Alfaro-Ruiz, Alfredo Hidalgo-Miranda, Vicente Madrid-Marina, Marcela Lizano

**Affiliations:** 1 Programa de Doctorado en Ciencias Biomédicas, Instituto de Investigaciones Biomédicas, Universidad Nacional Autónoma de México, Circuito Escolar S/N, Ciudad Universitaria, Delegación Coyoacán, 04500 Mexico City, Mexico; 2 Unidad de Investigación Biomédica en Cáncer, Instituto Nacional de Cancerología, México/Instituto de Investigaciones Biomédicas, Universidad Nacional Autónoma de México, Av. San Fernando No. 22, Col. Sección XVI, Tlalpan, 14080 Mexico City, Mexico; 3 Cátedras CONACyT-Instituto Nacional de Cancerología, San Fernando No. 22, Col. Sección XVI, Tlalpan, México City, Mexico; 4 Laboratorio de Genómica del Cáncer, Instituto Nacional de Medicina Genómica, México City, Mexico; 5 Dirección de Infecciones Crónicas y Cáncer. Centro de Investigación sobre Enfermedades Infecciosas (CISEI), Instituto Nacional de Salud Pública, Av. Universidad 655, Santa María Ahuacatitlán, Cuernavaca, Morelos, 62100 Mexico; 6 Departamento de Medicina Genómica y Toxicología Ambiental, Instituto de Investigaciones Biomédicas, Universidad Nacional Autónoma de México, 04510 Ciudad de México, Mexico

**Keywords:** Tumour virus infections, Human papilloma virus

## Abstract

The Human Papillomavirus (HPV) E1 protein is the only viral protein with enzymatic activity. The main known function of this protein is the regulation of the viral DNA replication. Nevertheless, it has been demonstrated that the ablation of HPV18 E1 mRNA in HeLa cells promotes a deregulation of several genes, particularly those involved in host defense mechanisms against viral infections; however, the specific contribution of E1 protein in HPV-independent context has not been studied. The aim of this work was to determine the effect of the HPV E1 protein in the regulation of cellular gene expression profiles evaluated through RNA-seq. We found that E1 proteins from HPV16 and 18 induced an overexpression of different set of genes associated with proliferation and differentiation processes, as well as downregulation of immune response genes, including IFNβ1 and IFNλ1 and Interferon-stimulated gene (ISG), which are important components involved in the antiviral immune response. Together, our results indicate that HR-(High-Risk) and LR-(Low-Risk) HPV E1 proteins play an important role in inhibiting the anti-viral immune response.

## Introduction

Infection with Human Papillomavirus (HPV) is the most frequent sexually transmitted disease worldwide, affecting more than a half of the sexually active population^1^. To date, close to 300 types of HPV have been identified based on their genomic sequences and approximately 40 of those types are able to infect the anogenital region^2^. HPVs are classified into high and low risk according to their oncogenic potential. Low-risk HPVs (LR-HPV) are associated with the development of genital warts, respiratory papillomatosis and low-grade cervical lesions, being the HPV6 and 11 the most prevalent types in such diseases^3^. High-risk HPVs (HR-HPV) are associated with the development of different types of anogenital cancers such as cervix, vulva, vagina, penis and anus, where HPV16 and 18 types are the most prevalent in cervical cancer, accounting for almost 70% of the cases. In addition, HR-HPVs have been associated with a subset of head and neck cancers, mainly with oropharyngeal cancer^4,5^.

The HPV genome contains an early expressed region containing the ORF (Open reading frames) of E1 to E7 genes which are necessary for viral replication and transcriptional regulation^6^. The E6, E7 and E5 proteins are able to interact with many cell targets, promoting cellular transformation^7,8^. The E1 protein is encoded within the early expressed region and it is localized in nuclear and cytoplasmic fractions. This protein is highly conserved among different HPV types and is the unique HPV protein with enzymatic activity^9^. The aminoacidic sequence of the E1 protein has been divided into three functional domains: the N-terminal domain, that contains a nuclear localization (NLS) and a nuclear export signal (NES); the central portion that harbors a DNA binding domain (DBD) and recognizes the replication origin within the LCR; and finally, the C-terminal domain, which has helicase/ATPase activity, as well as sequences involved in protein oligomerization^9,10^. The main function attributed to the E1 protein is its participation in viral DNA replication^11^. Viral genome replication depends on the ability of E1 to interact with several cellular proteins of the replication machinery such as α-DNA polymerase, replication A proteins (RPA), topoisomerases I and II, nuclear proliferation cell antigen (PCNA) and the replication factor C (RFC)^9^. However, it has been shown that E1 also interacts with other cellular elements independently of their function in viral replication, mainly with those that regulate epigenetic processes, such as histone H1 and Ini1/hSNF5 which is a subunit of the chromatin remodeling complex SWI/SNF, favoring the viral replication^12,13^.

Previous studies identified the presence of the HPV18 E1 protein in HeLa cells^14^. Afterwards, it was demonstrated that knocking down the expression of E1 by siRNAs, de-regulates gene expression, particularly four sets of genes involved in host defense mechanisms against viral infections including TLR signaling, interferon signaling, antiviral interferon stimulated genes (ISG) and apoptosis signalling^15^. Therefore, the aim of this study was to further analyze if E1 proteins from low and high-risk HPVs affect gene expression profiles. RNA-seq analysis showed that E1 proteins from HPV16, 18 and 11 differentially regulate gene expression profiles. Particularly, E1 from these three viral types down-regulated genes associated with the immune response. In addition, all E1 proteins were able to inhibit the expression of IFNβ1 and IFNλ1, two key components in the antiviral immune response. Our results suggest that E1 proteins regulate immune response gene expression which is shared among high- and low-risk HPV types.

## Results

### HPV E1 proteins regulate cellular gene expression

**Figure 1:**
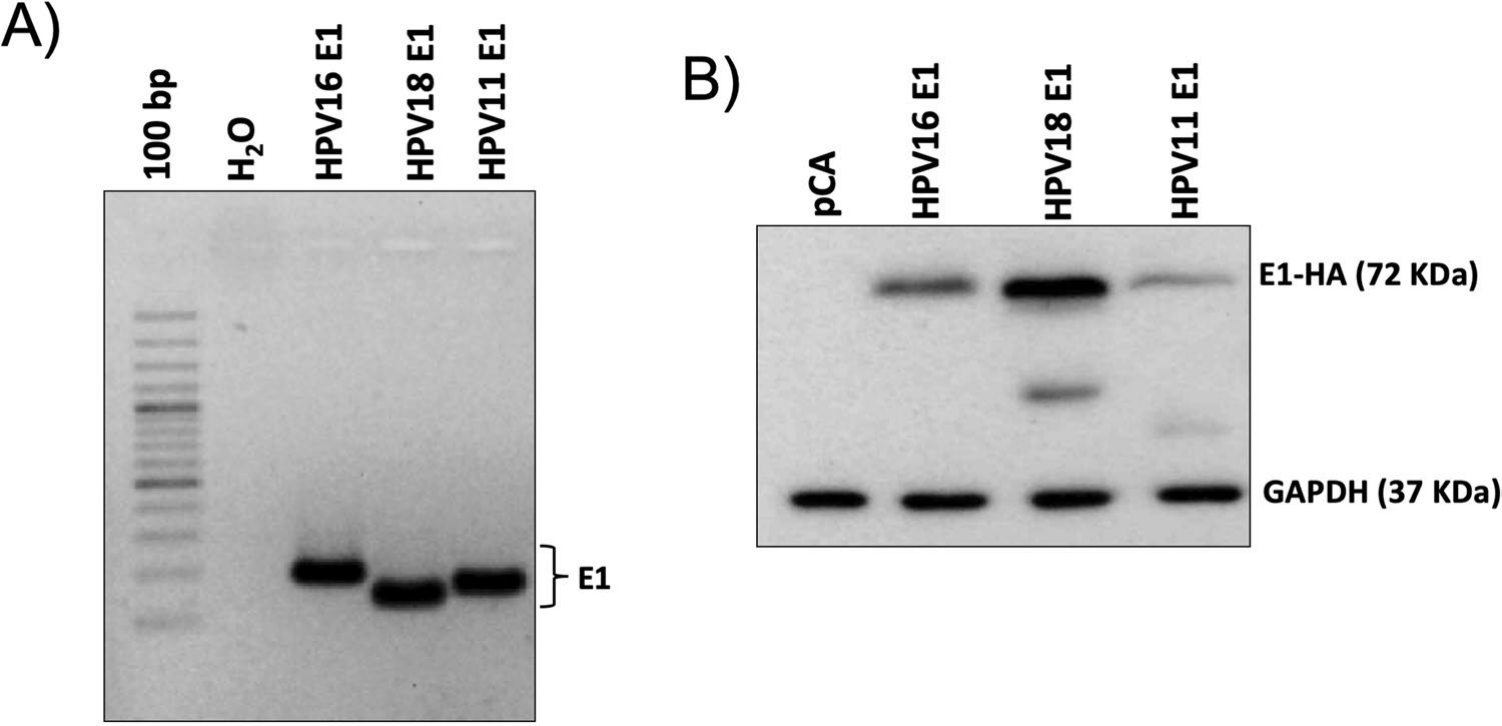
HA-HPV E1 expression in HaCaT cells. (**A**) HPV16, 18 and 11 E1 mRNA expression. HaCaT cells transfected with the pCA control vector and the HPV E1 expressing plasmids were collected 24 h after transfection. Then RNA was extracted, cDNA synthesized, and PCR performed to evaluate E1 mRNA expression. Amplification of a fragment of the E1 mRNA indicates that all three plasmids were successfully expressed in HaCaT cells. (**B**) HA-tagged HPV E1 protein expression. HaCaT transfected cells were collected and protein expression ascertained by western blot using anti-HA antibody. HPV16, 18 and 11 E1 HA tagged proteins were detected at 72 KDa. Additional bands are part of the E1 protein processing. Glyceraldehyde 3 phosphate dehydrogenase (GAPDH) was used as a loading control (37 KDa).

In order to analyze the effect of HPV E1 from low and high-risk HPVs on cellular gene expression profiles, an RNA-Seq analysis was performed from HaCaT cells expressing the HPV E1 proteins from three viral types. E1 expression from HPV tested types was evaluated 24 h post-transfection by RT-PCR and western blot as shown in Fig. 1A,B, respectively. Western blot analysis detected the E1 protein at approximately 72 KDa as well as other bands produced by the E1 cleavage according to the reported by Moody *et al*.^16^. Complete blot of the membrane is displayed in Supplementary Fig. 1.

Once E1 expression was confirmed, RNA-Seq was carried out using RNA coming from HaCaT cells transfected with the different HPV E1 expressing plasmids, as well as the pCA vector. Data analysis was performed considering a *p*-value of ≤0.05 as significant. Based on the log 2-fold change values, we found that HPV16 E1 up-regulates 199 genes and down-regulates 103 genes while HPV18 E1 up-regulates 45 genes and down-regulates 41, and HPV11 E1 up-regulates 34 genes and down-regulates 38 (Additional files 1, 2 and 3). When analyzing the cellular process affected by the E1 deregulated genes, we found that HPV E1 affects genes involved mainly in immune response as well as viral genome regulation (Table 1). These results indicate that the E1 protein from both, high and low risk HPVs have an effect on different cellular processes but mainly associated with immune response.

**Table 1:** Cellular processes affected by the deregulated genes induced by HPV E1 proteins. p-value ≤ 0.05.

HPV16 E1		HPV18 E1		HPV11 E1	
Cellular process	% terms per group	Cellular process	% terms per group	Cellular process	% terms per group
Regulation of peptidyl-tyrosine phosfhorylation	27.78%**	Endothelial cell chemotaxis	29.95%**	Regulation of MHC class I biosynthetic process	31.82%**
Formation of primary germ layer	16.67%**	Negative regulation of viral genome replication	18.42%**	Melanosome localization	27.73%**
Cellular response to vascular endothelial growth factor stimulus	16.67**	Negative regulation of viral genome transcription	15.79%**	MDA-5 signaling pathway	13.64%**
Negative regulation of viral genome replication	16.67**	Positive regulation of epidermal cell differentiation	13.16%**	Regulation of natural killer cell chemotaxis	11.36%*
Defense response to virus	5.56%**	Regulation of toll-like receptor 9 signaling pathway	7.89%**	Viral genome replication	11.36%**
Cellular response to interferon-gamma	5.56%**	Monocyte differentation	5.26%**	Type I interferon signaling pathway	4.55%**
Cytokine-mediated signaling pathway	5.56%**	MDA-5 signaling pathway	5.26%**	Pharyngeal system development	2.27%*
Regulation of bone mineralization	5.56%**	Cellular response to corticotropin-releasing hormone stimulus	2.63%**	Bile acid and bile salt transport 2.27%*	2.27%*
		Response to corticosterone	2.63%**		

**Figure 2:**
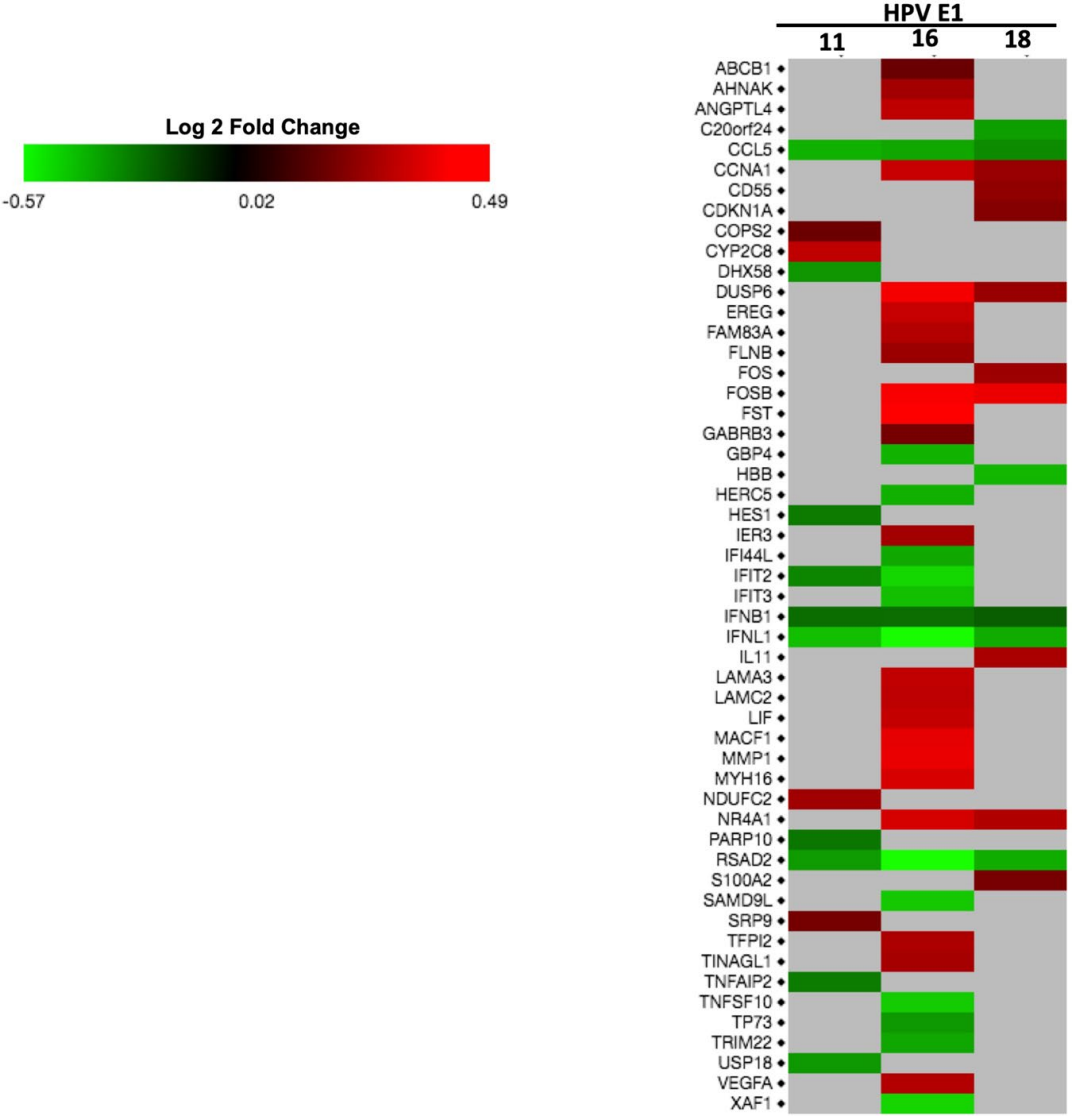
Genes differentially regulated by HPV E1 proteins. Heatmap showing genes differentially expressed in presence of HPV16, 18 and 11 E1 proteins based on the p-adj value (≤0.05). The scale of Log 2-fold change indicates the expression level of the genes. Red indicates overexpressed genes (expression levels over the median), green indicates under-expressed genes (expression levels under the median; see legend), and grey indicates unchanged genes.

**Figure 3:**
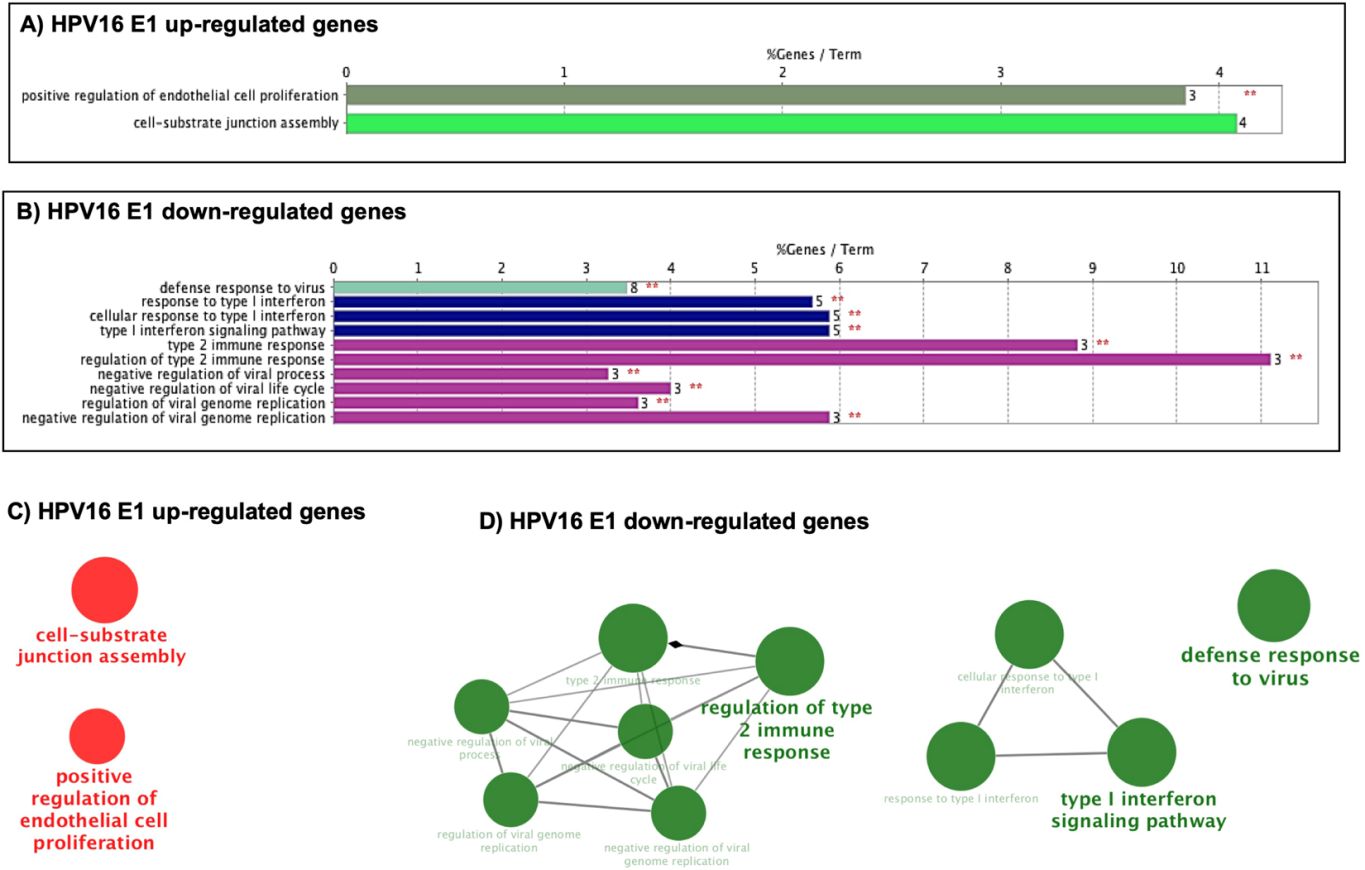
Immune response, cell-substrate junction assembly and cell proliferation networks are altered by the HPV16 E1 protein. HPV16 E1 significantly regulated genes were analyzed by ClueGO. (**A**) Gene ontology (GO) pathway terms obtained for up-regulated genes. (**B**) GO pathway terms obtained for down-regulated genes. The number of associated genes is depicted on the right of each bar and above is presented the percentage of altered genes. Significance (p ≤ 0.05) is represented by red asterisks. Clustered networks for up-regulated genes (Red color) (**C**) and downregulated genes (Green color) (**D**) in presence of HPV-16 E1 protein. The major term per group is displayed. Node size exposes the degree of enrichment. Organic layout algorithm introduced in Cytoscape supplied the obtained networks. Genes showing Padj changes with a significance of p < 0.05 were analyzed.

**Figure 4:**
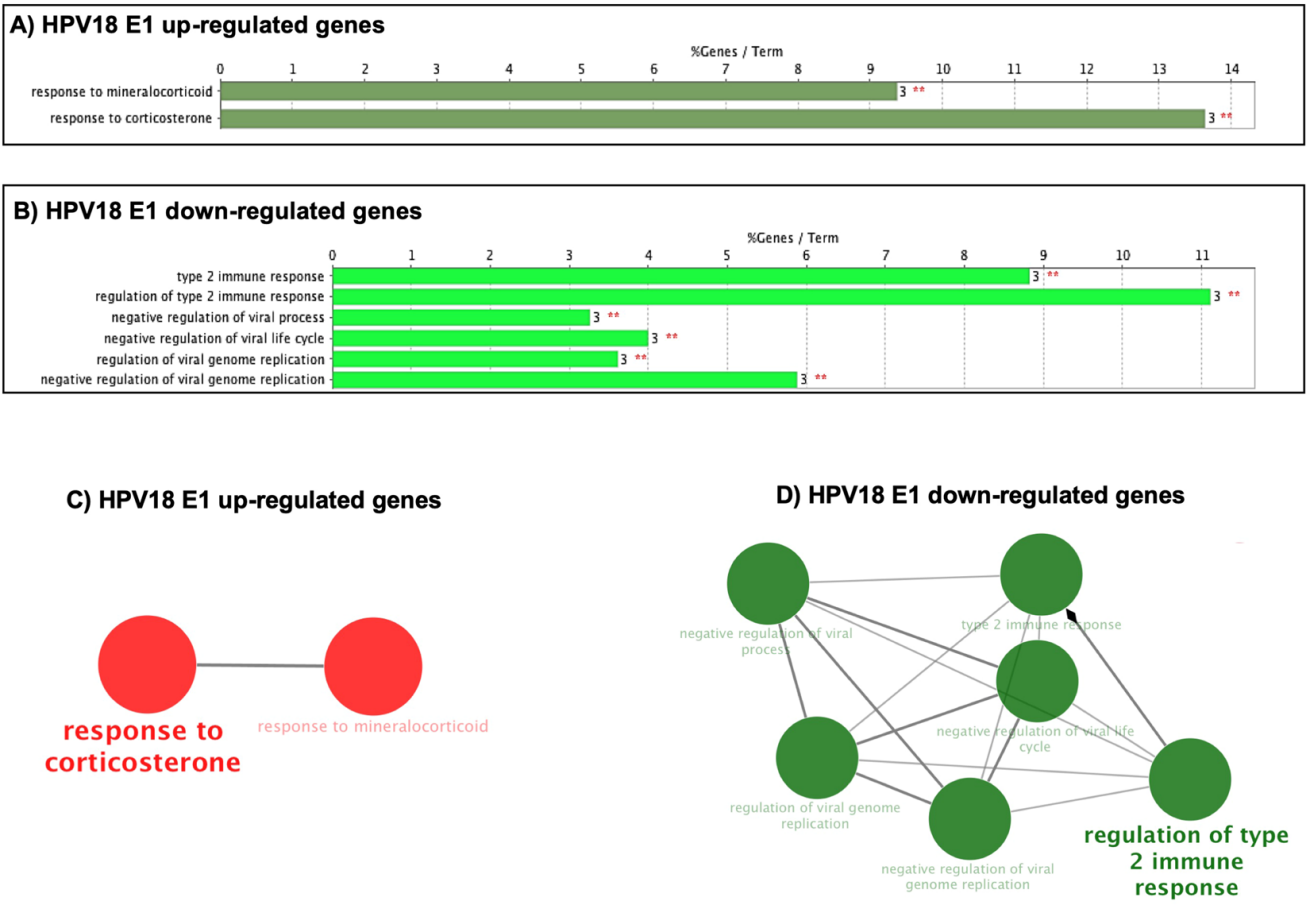
Immune response and response to corticosterone networks are altered by the HPV18 E1 protein. HPV18 E1 significantly regulated genes were analyzed by ClueGO. (**A**) Gene ontology (GO) pathway terms obtained for up-regulated genes. (**B**) GO pathway terms obtained for down-regulated genes. The number of associated genes is depicted on the right of each bar and above is presented the percentage of altered genes. Significance (p ≤ 0.05) is represented by red asterisks. Clustered networks for up-regulated genes (Red color) (**C**) and downregulated genes (Green color) (**D**) in presence of HPV-18 E1 protein. The major term per group is displayed. Node size exposes the degree of enrichment. Organic layout algorithm introduced in Cytoscape supplied the obtained networks. Genes showing Padj changes with a significance of p < 0.05 were analyzed.

**Figure 5:**
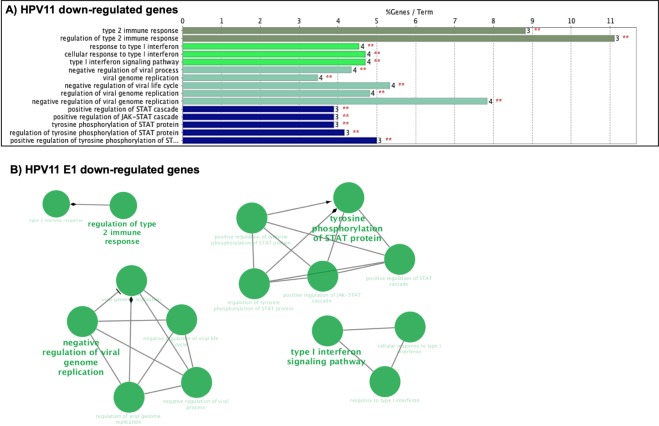
Immune response network is altered by the HPV11 E1 protein. HPV11 E1 significantly regulated genes were analyzed by ClueGO. (**A**) Gene ontology (GO) pathway terms obtained for down-regulated genes. The number of associated genes is depicted on the right of each bar and above is presented the percentage of altered genes. Significance (p ≤ 0.05) is represented by red asterisks. (**B**) GO pathway terms obtained for down-regulated genes. Clustered networks for downregulated genes (Green color) in presence of HPV-11 E1 protein. The major term per group is displayed. Node size exposes the degree of enrichment. Organic layout algorithm introduced in Cytoscape supplied the obtained networks. Genes showing Padj changes with a significance of p < 0.05 were analyzed.

To further dissect the effect of E1 on the expression of cellular genes, a second analysis was made to considering only the genes that were significant based on the adjusted p-value (p-adj) of ≤0.05. The results indicated that HPV16 E1 modulates 36 genes; while 15 and 14 genes were regulated by E1 HPV18 and HPV 11, respectively (Table 2). Based on the log 2-fold change values, we found that HPV16 E1 up-regulates 22 genes and down-regulates 14 genes, HPV18 E1 up-regulates 9 genes and down-regulates 6 genes; and HPV11 E1 up-regulates 4 genes and down-regulates 10 genes (Fig. 2). To analyze the cellular processes in which genes affected by E1 are involved, a Gene Ontology analysis (ClueGO) was performed. Supporting the previous results, our data revealed that HPV16 E1 up-regulated genes that participate in immune response, cell-substrate junction assembly and cell proliferation (Fig. 3A,C). Moreover, HPV16 E1 down-regulated genes involved in immune response processes such as response to type I interferon and regulation of type 2 immune response (Fig. 3B,D). Meanwhile, Gene Ontology analysis revealed that genes up-regulated by HPV18 E1 are involved in response to mineralocorticoid and corticosterone (Fig. 4A,C), whilst down-regulated genes are related to processes associated with regulation of type 2 immune response (Fig. 4B,D). Finally, when analyzing genes de-regulated by the HPV11 E1 protein, no cellular processes were associated to the up-regulated genes, probably because of the small number of genes (four genes) compared to those up-regulated by the high-risk HPVs. In contrast, the ClueGO program showed that down-regulated genes by HPV11 E1 are involved in processes such as the negative regulation of viral genome replication, tyrosine phosphorylation of the STAT protein, type I interferon signaling pathway and regulation of type 2 immune response (Fig. 5A,B).

**Table 2:** Differentially expressed genes by HPV E1 proteins.

	Number of genes p-adj	Genes
HPV16 E1	36	*FST*, *FOSB*, *DUSP6*, *MMP1*, *MACF1*, *MYH16*, *NR4A1*, *CCNA1*, *EREG*, *LIF*, *LAMA3*, *ANGPTL4*, *LAMC2*, *FAM83A*, *VEGFA*, *TFPI2*, *TINAGL1*, *AHNAK*, *IER3*, *FLNB*, *GABRB3*, *ABCB1*, *IFNB1*, *TP73*, *TRIM22*, *IFI44L*, *CCL5*, *HERC5*, *GBP4*, *IFIT3*, *SAMD9L*, *TNFSF10*, *XAF1*, *IFIT2*, *IFNL1*, *RSAD2*
HPV18 E1	15	*FOSB*, *NR4A1*, *IL11*, *DUSP6*, *FOS*, *CCNA1*, *CD55*, *CDKN1A*, *S100A2*, *IFNB1*, *CCL5*, *C20orf24*, *IFNL1*, *RSAD2*, *HBB*
HPV11 E1	14	*CYP2C8*, *NDUFC2*, *SRP9*, *COPS2*, *IFNB1*, *PARP10*, *HES1*, *TNFAIP2*, *IFIT2*, *DHX58*, *USP18*, *RSAD2*, *CCL5*, *IFNL1*

Cellular genes with differential expression detected by RNA-Seq from 24 h post-transfected HaCaT cells. Adjusted p-value ≤ 0.05.

### The E1 proteins from low- and high-risk HPVs regulate sets of genes in common

**Figure 6:**
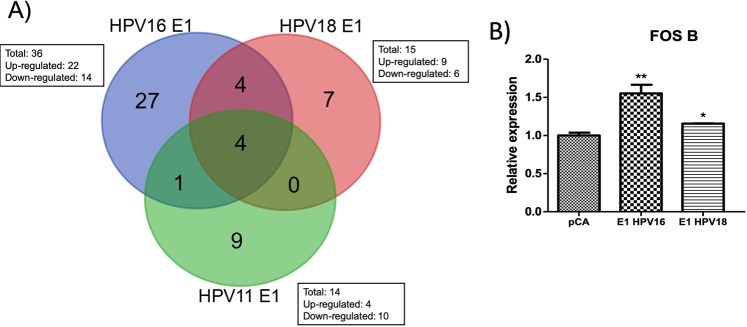
The HPV16, 18 and 11 E1 proteins alter similar genes. (**A**) Venn diagram summarizing the shared number of genes overlapping between differentially expressed genes altered by HPV E1 proteins. (**B**) HaCaT cells transfected with pCA vector control and HPV16 and HPV18 E1 expressing plasmids were collected and RNA extracted 24 h after transfection. Then, cDNA was synthesized and the mRNA levels of FOSB was measured using specific primers. Relative mRNA levels of FOSB showed an increase in presence of E1 from HPV16 and 18. Values are expressed as the difference in double delta-Ct compared with pCA control transfected cells. The expression of the housekeeping gene 18S was used for normalizing. Bars represent the mean ± SD. *p < 0.05 *vs* control (pCA).

We determined whether genes altered by E1 proteins are shared among the different HPV types evaluated. Venn diagram, from the obtained data shows that HPV16 and HPV18 E1 proteins deregulated four shared genes: *FOSB*, *DUSP6*, *NR4A* and *CCA1* (Fig. 6A). The expression of FOSB was verified by qRT-PCR (Fig. 6B), finding increased mRNA levels in the presence of E1 from HR-HPVs compared to the pCA expressing cells (p < 0.001).

HPV16 and HPV11 types shared only one gene (*IFIT2*). It was also found that E1 from HPV18 and HPV11 do not share de-regulated genes. Interestingly, four immune response genes are deregulated by the three HPV types (*IFNB1*, *IFNL1*, *CCL5 or RANTES*, and *RSAD2 or Viperin*) (Fig. 6A). This suggests that the HPV E1 proteins might have a high impact on the expression of genes associated with the immune response. In addition, it shows that this effect is shared between high and low risk HPVs.

### E1 proteins from HPV16, 18 and 11 decrease IFNβ1 and IFNλ1 expression

**Figure 7:**
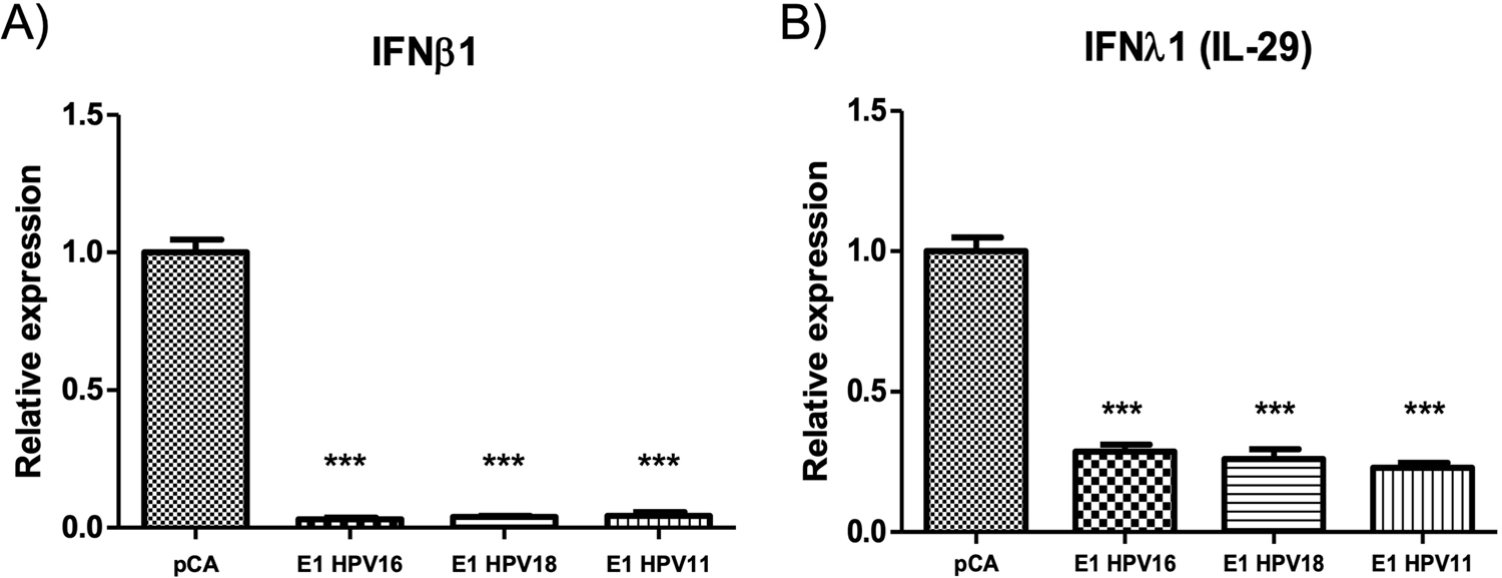
The E1 protein from HPV16, 18 and 11 downregulates IFNβ1 and IFNλ1 expression. HaCaT cells transfected with pCA control vector and HPV E1 expressing plasmids were collected and RNA extracted 24 h after transfection. Then, cDNA was synthesized and the mRNA levels of IFNβ1 and IFNλ1 genes were measured using specific primers (Supplementary Table 1). Relative mRNA levels of (**A**) IFNβ1 and (**B**) IFNλ1 genes showing a decrease in presence of the HPV-16, 18 and 11 E1. Values are expressed as the difference in double delta-Ct compared with pCA control transfected cells. The expression of the housekeeping gene 18S was used for normalizing. Bars represent the mean ± SD. ***p < 0.0001 *vs* control (pCA).

**Figure 8:**
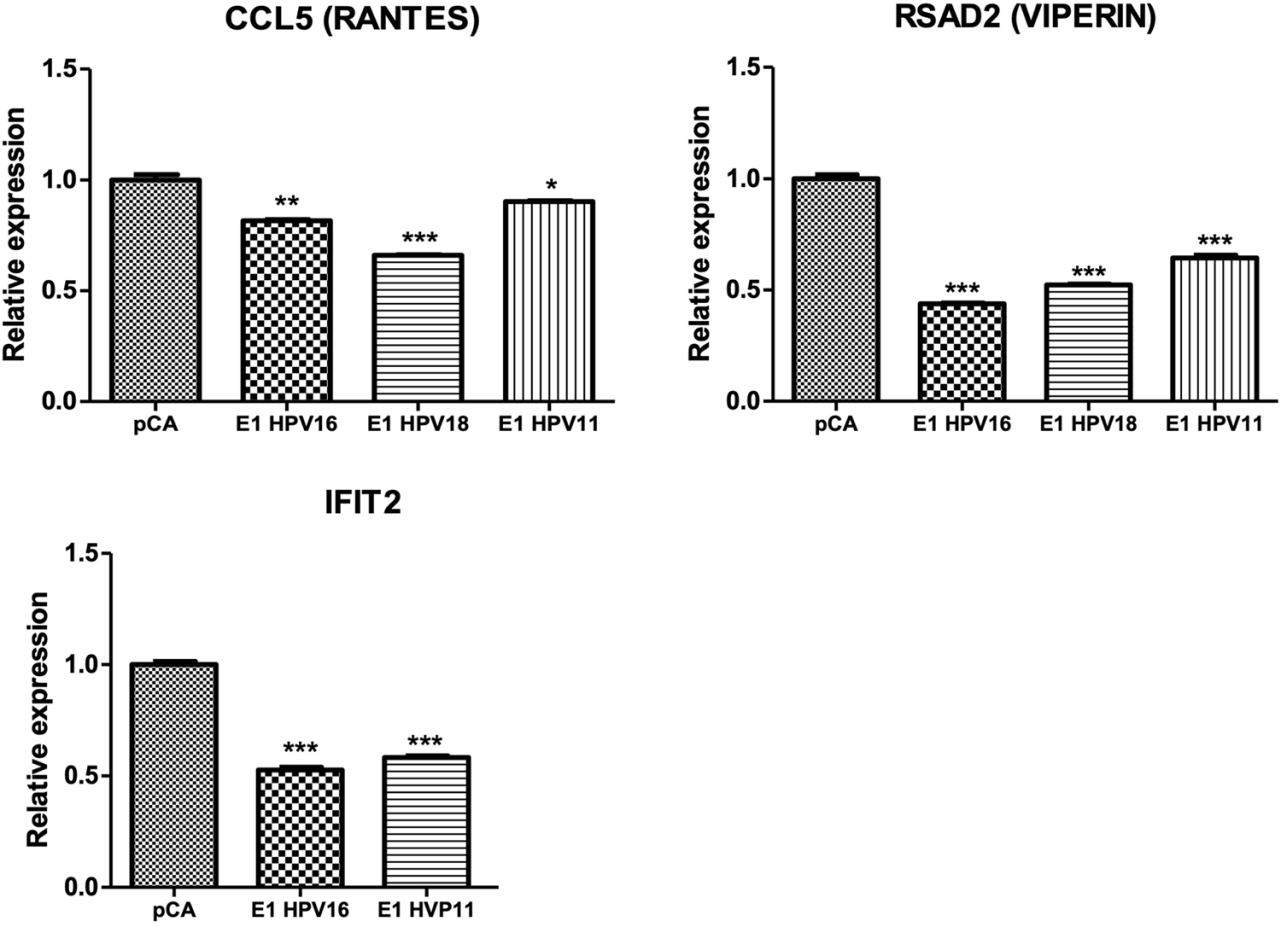
The E1 protein from HPV16, 18 and 11 downregulates interferon-stimulated genes expression. HaCaT cells transfected with pCA vector control and HPV E1 expressing plasmids were collected and RNA extracted 24 h after transfection. Then, cDNA was synthesized and the mRNA levels of CCL5, RSAD2 (Viperin) and IFIT2 genes were measured using specific primers (Supplementary Table 1). Relative mRNA levels of CCL5, RSAD2 (Viperin) and IFIT2 genes showing a decrease in presence of the HPV-16, 18 and 11 E1. Values are expressed as the difference in double delta-Ct compared with pCA control transfected cells. The expression of the housekeeping gene 18 S was used for normalizing. Bars represent the mean ± SD. *p < 0.05, **p < 0.01, ***p < 0.001 *vs* control (pCA).

As it was shown, HPV E1 proteins induced a down-regulation in the expression of four genes involved in immune response (*CCL5, RSAD2, IFNB1 and IFNL1*). Due to their important role in the viral innate immune response^17^, both IFNβ1 and IFNλ1, were selected to perform further analyses. IFNβ1 and IFNλ1 promote the expression of ISG genes that help to control infectious processes allowing an efficient antiviral immune response^18^. Expression of IFNβ1 and IFNλ1 was validated through qPCR assays in HaCaT cells expressing the different E1 proteins. Interestingly, E1 viral proteins decreased IFNβ1 (Fig. 7A) and IFNλ1 (Fig. 7B) mRNA levels compared to the control vector (p < 0.001). Since the all three HPV types affected the expression of ISGs, we next tested the mRNA levels of CCL5 and RSAD2 (viperin) finding a significant decrease in presence of the three E1 proteins compared to pCA expressing cells (p < 0.001) (Fig. 8). Additionally, the mRNA levels of IFIT2, the only gene shared between HPV16 and HPV11 types, were significantly diminished (p < 0.001) by both E1 proteins (Fig. 8).

**Figure 9:**
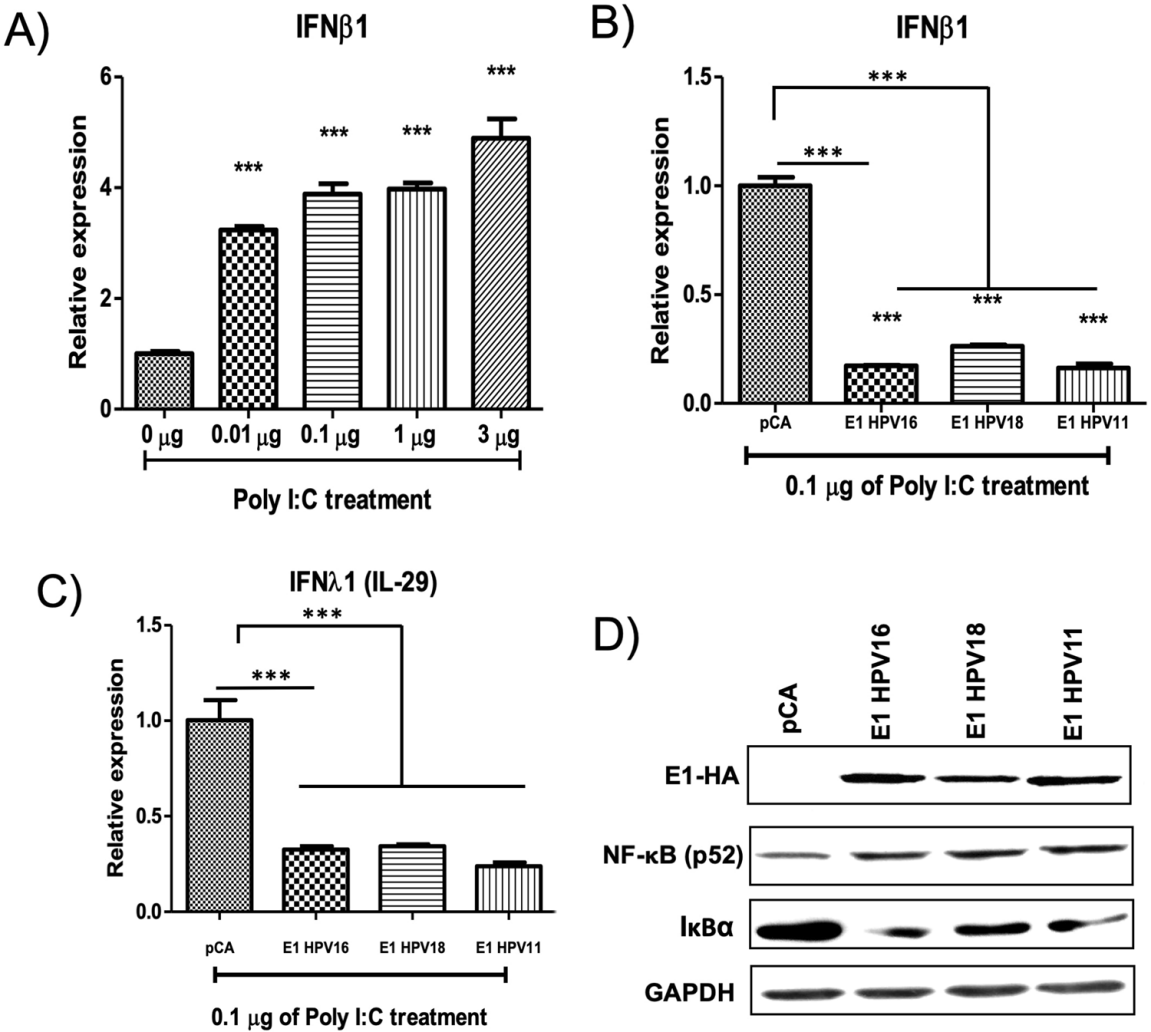
HPV E1 protein decreases IFNβ1 and IFNλ1 expression in the presence of the Poly I:C agonist. HaCaT cells were transfected using 0.01, 0.1, or 1 μg of Poly I:C and pCA vector to a final amount of 4.5 μg. After 24 h post transfection total RNA was extracted and the IFNβ1 expression was evaluated by pPCR. (**A**) The Poly I:C increases the IFNβ1 and IFNλ1 expression in HaCaT cells in all concentrations tested. The 0.1 μg concentration was selected for further analyses. Bars represent the mean ± SD. ***p < 0.001 *vs* control (pCA). (**B**) IFNβ1 and (**C**) IFNλ1 expression is repressed in the presence of HPV16, 18 and 11 E1 proteins. After 24 h of transfection with 0.1 μg of Poly I:C and the indicated HPV E1 plasmid, total RNA was extracted, cDNA synthesized, and qPCR performed to evaluate the IFNβ1 and IFNλ1 expression. In presence of the different HPV E1 proteins there is a decrease of IFNβ1 and IFNλ1 expression. Values are expressed as the difference in double delta-Ct compared with cells transfected with the pCA vector. The expression of the housekeeping gene 18S was used for normalizing RNA expression. Bars represent the mean ± SD. ***p < 0.001 *vs* pCA control. (**D**) IkBα and p52-NFκB protein levels. HaCaT cells transfected with pCA vector and HPV E1 expressing plasmids were collected and total protein extracted 24 h after transfection. Levels of IkBα and p52-NFκB were ascertained by western blot. Proteins were separated in a 10% SDS-PAGE and membrane was probed with anti-phospho-IκBα (Ser32/36)(5A5) (9246; Cell signaling Technology Europe, B.V) or anti-p52 (NFκB) (Santa Cruz Biotechnology, California) observing decreased levels of IkBα and an increase of p52-NFκB levels in the presence of HPV16, 18 and 11 E1 proteins. GAPDH was used as loading protein control (GAPDH, 37 KDa).

Since HPV E1 proteins inhibit the expression of IFN, we wonder if E1 could interfere with the production of IFN after stimulating with an inductor of IFNβ. Poly I:C is a synthetic analog of viral dsRNA and it is a well-established inductor of the IFNβ production^19,20^. Increasing amounts of Poly I:C were transfected (0.01, 0.1, 1 and 3 μg) in order to induce IFNβ expression in HaCaT cells. At 24 h post-transfection, total RNA was extracted and levels of IFNβ1 mRNA were evaluated by qPCR. Results indicate that IFNβ1 expression significantly increases in cells transfected from 0.01 to 3 μg of Poly I:C (Fig. 9A) (p < 0.0001).

A previous report showed that 0.1 μg of Poly I:C was effective for stimulation and those amounts did not affect the viability of HaCaT cells at 24 hours^21^; therefore, 0.1 μg was selected to perform subsequent experiments. To determine whether E1 interferes with IFNβ1 expression during Poly I:C treatment, HaCaT cells were co-transfected with 0.1 μg of Poly I:C and 4 μg of each HPV E1-expressing plasmid or the control plasmid. After 24 h post-transfection, expression of IFNβ1 was evaluated by qPCR. Figure 9B shows that cells expressing HPV16, 18 and 11 E1 proteins exhibit a decrease in the levels of IFNβ1 compared with cells transfected only with poly I:C plus the pCA vector (p < 0.0001). Additionally, it was demonstrated that 0.1 μg of Poly I:C promoted the expression of IFNλ1 mRNA in the presence of the control vector; while such expression decreased in E1-expressing cells (p < 0.0001) (Fig. 9C). Taken together, these results demonstrate that HPV E1 proteins from low- and high-risk decrease the expression of IFN even after stimulating IFN gene transcription, showing that HPV16, 18 and 11 E1 proteins downregulate the expression of two key components of the antiviral immune response.

In order to verify the possible mechanism implicated in the downregulation of IFNs induced by the HPV E1 proteins, we evaluated the levels of NFκB, a master regulator of immune related genes^22^, as well as the levels of IkBα, which is a negative regulator of NFκB. As shown in Fig. 9D, HPV E1 proteins increased p52 (NFκB) protein levels, which is mainly a positive regulator of the NFκB non-canonical pathway. Additionally, in the presence of E1 proteins, IκBα (NFκB canonical pathway negative regulator) protein levels decreased. These results indicate that HPV E1 activates not only the NFκB canonical pathway, but also the non-canonical pathway.

## Discussion

The function so far attributed to the HPV E1 protein has been its participation in the viral replication process, along with E2^11^. However, it has been shown that E1 also associates with other cellular proteins that regulate epigenetic processes^12,13,23^. Thus, the ability of E1 to interact with chromatin modifiers to regulate viral replication, suggests that this protein could also affect cellular processes in the host genome such as gene expression.

In the present study, we determined the effect of E1 from high and low risk HPVs on cellular gene expression analyzed by RNA-seq observing that E1 protein affects the expression of different cellular processes, however, a greater effect on cellular processes associated with immune response (p-value < 0.05) was observed. More restricted analysis in which only genes with an adjusted p-value (p-adj < 0.05) were selected, identified a smaller number of genes regulated by the presence of E1, which are involved in immune response more specifically in antiviral immune response. Our results are in agreement with those of Castillo *et al*., (2014) who reported a change in the expression of genes associated with the innate immune response after the ablation of E1 expression in HeLa cells^15^. Furthermore, when comparing the three viral types tested, our results demonstrated that not only E1 from HPV18 promotes a change in gene expression but also E1 from HPV16 and from low-risk HPV 11, suggesting a probable similar mechanism in the regulation of gene expression performed by the E1 proteins. Particularly, 4 genes were downregulated in common by the three HPV E1 proteins (IFNβ1, IFNλ1, Viperin and CCL5), which participate in the antiviral immune response^17,24–26^.

IFNβ1 and IFNλ1, are key mediators of the antiviral immune response, which ultimately promote the expression of interferon-stimulated (ISG) genes, helping to control the infectious processes, through multiple mechanisms such as protein translation inhibition, viral RNA degradation as well as the activation and survival of innate and adaptive immune cells such as Dendritic cells (DCs), macrophages, NK cells, and T cells, activating both innate and adaptive immune response to inhibit viral infection^18,27,28^. Due to the relevance of such elements, IFNβ1 and IFNλ1 were selected for further validation through qPCR, that demonstrated that mRNA levels decreased in the presence of the three viral types. Importantly, the E1 proteins blocked the IFNβ expression even after stimulating with Poly I:C which produces a similar effect than a viral infection, indicating that the downregulation induced by the E1 proteins at a transcriptional level has an impact during a normal challenge in immortalized keratinocytes. Similarly, when analyzing IFNλ1, we found that E1 also decreased its expression after Poly I:C treatment, suggesting that E1 could be acting as an immunosuppressive element in HPV infections. The fact that E1 decreases IFNβ1 and IFNλ1 mRNA levels, lead us to evaluate the expression of some ISGs, finding a decrease in the expression of CCL5, RSAD2 (viperin) and IFIT2 in the presence of E1 of HPV16, 18 and 11. Such results support the data obtained by RNA-seq, in which these ISGs were also diminished, demonstrating that E1 affects the expression of IFNs, which in turn affects the expression of ISGs. Thus, suggesting that E1 may play an important role in the antiviral immune response.

We found a reduction of the negative regulator IκBα in the presence of the three HPV E1 proteins, indicating that the NFκB canonical pathway is active. These results are in agreement with those reported by Nakahara *et al*., (2015) where HPV16 E1 induced the activation of NFκB in primary cervical keratinocytes^29^. To test whether E1 could activate the NFκB non-canonical pathway, we analyzed p52 levels, a product of NF-κB2/p100 processing. It is known that p52 associates with RelB in the cytoplasm and the complex RelB:p52 is required for the induction of several pro-inflammatory genes^30^. Interestingly, we found an increase in p52 levels in the presence of the three HPV E1 proteins. These results suggest that HPV E1 protein is somehow activating the two NFκB pathways promoting the expression of genes such as cytokines, chemokines, adhesion molecules, enzymes that produce secondary inflammatory mediators, and inhibitors of apoptosis^31^. Nevertheless, while the activation of NFκB should promote the expression of IFNβ we found that E1 decreases its expression. This contradictory effect could be due to the fact that NFκB is not essential for IFNβ expression^32^, although E1 could favor the expression of other NFκB genes. So it would be interesting to determine whether E1 affects other transcriptional factors or cofactors, that could regulate the expression of IFNβ, such as IRFs, since it has been shown that the Interferon Regulatory Factor 3 (IRF-3) promotes the expression of IFNβ^33^ as has been shown for E6 and E7 oncoproteins^34–36^.

That E1 blocks the IFNs production indicates that in addition to its participation in HPV infection, E1 could act on HPV persistency; nevertheless, the particular role of E1 in such process needs to be addressed. Therefore, it would be interesting to analyze the mechanism by which E1 interferes with the expression and production of IFN, as well as its effect on the expression of ISG. Some studies have demonstrated that the HPV produces an alteration of key immunological elements either in primary keratinocytes in a context of whole genome as well as in cell lines. Several of the altered genes found in this study were confirmed by those studies including the analysis of IFNβ and RANTES^37,38^, and ISGs^15^. It is worth mentioning that the inhibition of INF expression has been observed in several viral infections such as Hepatitis, Herpesvirus and HTLV-1, where viruses use this strategy to evade the innate immune response favoring the infectious process^39–41^. Our results propose a novel mechanism in the modulation of the immune response induced by the HPV E1 protein, which could be associated to the replicative cycle and the viral persistence by evading the immune response.

Among the processes associated with the development of cancer induced by HPV, viral persistence represents a leading key event at the beginning of this process^42^. Recently it was determined that E1 is expressed throughout the different stages of cervical cancer^43^. The authors analyzed the levels of HPV16 E1 mRNA in biopsies with different lesion degrees (CIN) as well as in cervical cancer, observing an increased expression of E1 as long as the lesion advances. In this regard, the analysis of genes differentially regulated by the E1 proteins, specifically those from the high-risk HPVs, revealed that E1 from both HPV16 and 18 types promote the expression of genes associated with cell proliferation, differentiation, cell viability and metabolism regulation (*FOSB, DUSP6, NR4A and CCNA1*)^44–46^, processes that could favor the replicative cycle. However, it has been demonstrated that overexpression of some of these genes play an important role in carcinogenesis associated with poor prognosis, resistance to treatment, recurrence and poor survival in some cancers (non-small-cell lung carcinoma, ovarian cancer)^46–48^. Moreover, E1 from HPV16 regulated the expression of genes such as *MMP1, EREG, ANGEPTL4* which regulate cellular processes such as keratinocyte migration and re-epithelialization, proliferation and angiogenesis^49,50^, interestingly, the overexpression of those genes has been associated with the carcinogenesis process^26,51–54^. Taken together, these data suggest that E1 could play a role in the development of HPV-related cancers through the overexpression of certain cellular genes.

During the carcinogenic process there exists a deregulation of gene expression and epigenetic mechanisms, which play an important role at the different stages of cancer; these mechanisms are capable of altering the organization of chromatin, through chromatin modifiers, favoring or inhibiting the expression of genes^55^. It is known that the HPV genome organizes itself into a chromatin like structure^56^, regulating viral replication and transcription, which requires the participation of chromatin modifiers. Also, it has been shown that E1 interacts with the histone H1 and with the Ini1/hSNF5^12,13^, which are involved in the regulation of gene expression. Therefore, the ability of E1 to interact with those proteins, as well as with other chromatin modifiers as acetyltransferases (HATs) and deacetylases (HDACs), could be one of the mechanisms implicated in the regulation of gene expression performed by the E1 protein. In addition, this suggests that E1 could not only regulate the HPV genome, but also the host genome, promoting gene expression that could be favoring in part the infectious process and probably carcinogenesis.

It is also possible that E1 regulates gene expression by directly binding to the cellular genome on regulatory elements (promoters or enhancers), through its DNA binding domain^57^. This has been demonstrated for the Epstein-Barr virus (EBV) EBNA1 protein, which directly bind to cellular promoters, which was related to changes in gene expression profiles^58^. However, it is likely that an indirect interaction of E1 with DNA through its interaction with transcription factors or with chromatin modifiers could be happening. Therefore, it would be interesting to study new E1 interacting partners that allow the modulation of gene expression, including the modulation of other cellular processes, such as its participation in cell transformation.

## Conclusions

Our results demonstrate that HPV E1 proteins induces changes in cellular gene expression, which is a process that is conserved among high- and low-risk HPVs. E1 of HPV16 and 18 are capable of regulating the expression of genes involved in processes of proliferation, migration and metastasis which are associated with carcinogenesis. Otherwise, E1 of the three viral types were able to modulate the expression of genes associated with the antiviral immune response, and also avoided the IFNβ1 and IFNλ1 expression, as well as of ISGs. Although the mechanism for such regulation remains unclear, our results suggest that both, the canonical, as well as the non-canonical NFκB pathways are affected by the HPV E1 proteins. Nevertheless, the participation of NFκB pathways in the regulation of IFN needs further investigation. Therefore, in addition to its well-known role in viral replication, the E1 protein may also play an important role in the evasion of the immune response to favor the replicative cycle, persistence and development of cancer.

## Materials and Methods

### Cell culture and transfections

The HPV negative cell line HaCaT, was kindly provided by Dr. A. Garcia-Carrancá (INCan, México). Cells were maintained in Dulbecco’s Modified Eagle’s Medium/Nutrient Mixture F-12 (DMEM/F-12) supplemented with 10% of foetal bovine serum (FBS). A total of 380,000 cells were plated in a 60 mm dish and transfected with the indicated plasmids using PolyFect® reagent (Qiagen, Hilden, Germany) according to the manufacturer’s instructions. For IFN expression stimulation, HaCaT cells were transfected with different amounts of Polyinosinic-polycytidylic acid (Poly I:C) (Sigma, St. Louis, MO, USA) as indicated. In each case, cells were collected and used for RNA and protein extraction after 24 h post transfection.

### Plasmids

HPV16 and HPV11 HA tagged E1 expressing plasmids were kindly provided from Dr. Mart Ustav (University of Tartu, Estonia). HPV18 HA tagged E1 expressing plasmid was previously described by our group^59^. The pCA vector was used as transfection control. All plasmids were verified by sequencing.

### RNA Isolation and cDNA synthesis

HaCaT cells were seeded in a 60 mm culture dish and transfected with 4.5 µg of each of the three viral types E1 plasmid in triplicate. After 24 h of transfection, total RNA was isolated from each condition using the RNeasy Mini kit (Qiagen, Hilden, Germany), according to the manufacturer’s protocol. RNA was resuspended in 30 μL of RNAse-free H_2_O, the was treated with the DNAse Free DNA removal kit (Thermo Fisher Scientific, Waltham, MA, USA) and quantified. 400 µg of RNA was reverse-transcribed with Oligo dT utilizing the GeneAmp RNA PCR Core Kit (Applied Biosystems, Foster City, CA, USA) The expression of E1 from the different HPVs were verified by RT-PCR using specific primers listed in Supplementary Table 1.

### RNA-Seq: Illumina library preparation and sequencing

Total RNA was isolated from three independent transfections per condition as indicated. Total RNA was quantified spectrophotometrically using a NanoDrop ND‐1000 spectrometer (Thermo Scientific, Waltham, MA, USA); integrity was analyzed by capillary electrophoresis using the Bioanalyzer 2100 (Agilent Technologies Inc., Santa Clara, CA, USA), all samples exhibited a high-quality RNA integrity number (RIN) > 9.0. RNA-seq libraries from all replicas and conditions (n = 12) were constructed using the TruSeq Stranded mRNA Library Preparation kit (Ilumina, San Diego, CA) as 76-bp paired-end reads length. Samples were sequenced using an Illumina NextSeq 500 platform, generating at least 40 million reads per sample. Paired-end reads were assigned quality scores and aligned to the reference genome RefSeq (hg19) using bowtie and then count files were generate with Htseq-count, and diferential expression performed with DESeq2 where *p*-value of < 0.05 and Adjusted *p*-value < 0.05 were consider as statistically significant. The pipepline was carried out using the tools provided in Illumina´s BaseSpace. Biological processes analysis (GO category) was performed using differentially expressed genes and visualized with ClueGo, a Cytoscape plug-in. Ingenuity pathway analysis was performed to identify canonical signaling pathways and functional pathways affected by the differentially expressed genes. Fisher’s exact test was done automatically by the software.

### Gene expression assay

Total RNA was isolated using the RNeasy mini kit (Qiagen, Hilden, Germany). The obtained RNA was treated with the DNAse Free DNA removal kit (Thermo Fisher Scientific, Waltham, MA, USA). A total of 500 µg of RNA was reverse-transcribed with Oligo (dt) primers utilizing the GeneAmp RNA PCR Core Kit (Applied Biosystems, Foster City, CA, USA). The expression of *FOSB*, *IFIT2*, *CCL5*, *RSAD2*, *IFNβ1* and *IFNλ1* genes was evaluated using specific primers listed in Supplementary Table 1. As a house keeping control, 18S gene expression was also evaluated. The results are presented as relative quantification using the ΔΔCt method.

### Western blot analysis

After 24 h post-transfection, cells were collected, and protein extracts obtained by adding Leammli sample buffer (Bio Rad, Ca, USA). Equal amounts of proteins were loaded and separated by SDS-PAGE and transferred onto a nitrocellulose membrane. The membrane was blocked using 7.5% milk in TBS/0.01% Tween buffer and then incubated with the indicated antibodies. Primary antibodies were prepared in TBS/0.01% Tween buffer as follows: anti-GAPDH (1:1000) (32233; Santa Cruz Biotechnology, Santa Cruz, California), anti-HA (1:1000) (C29F4; Cell Signaling Technology Europe, B.V), anti-p52NFκB (1:1000) (298; Santa Cruz Biotechnology, Santa Cruz, California), α-Actinin (1:1000) (17829; Santa Cruz Biotechnology, Santa Cruz, California) and Phospho-IκBα (Ser32/36)(5A5) (9246; Cell signaling Technology Europe, B.V) Membranes were washed three times with TBS/0.01% Tween buffer and incubated with HRP coupled secondary anti-mouse or anti-rabbit antibodies (Santa Cruz Biotechnology, Santa Cruz, California). Finally, Proteins were visualized using the ChemoLuminiscent Reagent (Merck Millipore, Burlington, Massachusetts, U.S) according to the manufacturer’s instructions.

### Statistical analysis

Data showing the effects of HPV E1 protein in the different assays are presented as mean ± SD. *p* was calculated by Student’s *t*-test or ANOVA Tukey’s post-hoc analysis. Significant differences were accepted with p ≤ 0.05, as indicated.

## Supplementary information

Supplementary table 1

Dataset 1

Dataset 2

Dataset 3
