# Supplementary material for: The Human Papillomavirus (HPV) E1 protein regulates the expression of cellular genes involved in immune response
Source: Sci Rep. 2019 Sep 20;9:13620. doi: 10.1038/s41598-019-49886-4 (PMC6754496; doi:10.1038/s41598-019-49886-4)
Supplement: Supplementary file 1 — Supplementary table 1 [file 41598_2019_49886_MOESM1_ESM.doc]

**The Human Papillomavirus (HPV) E1 protein regulates the expression of cellular genes involved in immune response**

Leonardo Josué Castro-Muñoz^1,2^, Joaquín Manzo-Merino^2,3^, J. Omar Muñoz-Bello^2^, Leslie Olmedo-Nieva^2^, Alberto Cedro-Tanda^4^, Luis Alberto Alfaro-Ruiz^4^, Alfredo Hidalgo-Miranda^4^, Vicente Madrid-Marina^5^ and Marcela Lizano^2,6^*

^1^Programa de Doctorado en Ciencias Biomédicas, Instituto de Investigaciones Biomédicas, Universidad Nacional Autónoma de México, Circuito Escolar S/N, Ciudad Universitaria, Delegación Coyoacán, 04500 Mexico City, Mexico.

^2^Unidad de Investigación Biomédica en Cáncer, Instituto Nacional de Cancerología, México/Instituto de Investigaciones Biomédicas, Universidad Nacional Autónoma de México, Av. San Fernando No. 22, Col. Sección XVI, Tlalpan, 14080 Mexico City, Mexico.

^3^CONACyT-Instituto Nacional de Cancerología, San Fernando No. 22, Col. Sección XVI, Tlalpan, México City, México.

^4^Laboratorio de Genómica del Cáncer, Instituto Nacional de Medicina Genómica. México City, México

^5^Dirección de Infecciones Crónicas y Cáncer. Centro de Investigación sobre Enfermedades Infecciosas (CISEI), Instituto Nacional de Salud Pública, Av. Universidad 655, Santa María Ahuacatitlán, Cuernavaca, Morelos, Mexico. 62100.

^6^Departamento de Medicina Genómica y Toxicología Ambiental, Instituto de Investigaciones Biomédicas, Universidad Nacional Autónoma de México, 04510 Ciudad de México, México.

*Author to whom correspondence should be addressed: lizanosoberon@gmail.com

**Supplementary Table 1.** List of primers used for PCR and the expected size of products.

| **Gene** | **Forward 5´** | **Reverse 3´** | **Amplified fragment length (bp)** |
| --- | --- | --- | --- |
| HPV16 E1 | GGTTACAACCATTAGCAGAT | ACCAATGGTCTATGCTTTAC | 200 |
| HPV18 E1 | CTGCTCACAACTAACATTCA | CCGTTCTTATCAAATGGGAA | 170 |
| HPV11 E1 | GTGTACAGGATGGTTTATGG | TGTCATCAATAAAGTCCACC | 150 |
| IFNβ1 | GCCAAGGAGTACAGTCACTG | TGAAGCAATTGTCCAGTCCC | 100 |
| IFNλ1 | CGCCTTGGAAGAGTCACTCA | GAAGCCTCAGGTCCCAATTC | 100 |
| 18s | AACCCGTTGAACCCATT | CCATCCAATCGGTAGTAGCG | 140 |
| CCL5 (RANTES) | GCTGTCATCCTCATTGCTACTG | TGGTGTAGAAATACTCCTTGATGT | 150 |
| RSAD2 (Viperin) | CACAAAGAAGTGTCCTGCTTGGT | AAGCGCATATATTTCATCCAGAATAAG | 150 |
| IFIT2 | ACTGCAACCATGAGTGAGAAG | GCCTCGTTTTGCCCTTTGAG | 140 |
